# Lyapunov Thermodynamic Stability of the Evolution of Conservatively Perturbed Chemical Equilibrium

**DOI:** 10.3390/e28020206

**Published:** 2026-02-11

**Authors:** Anil A. Bhalekar, Vijay M. Tangde, Bjarne Andresen

**Affiliations:** 1 Department of Chemistry, Rashtrasant Tukadoji Maharaj Nagpur University, Nagpur 440 033, India; anabha@hotmail.com (A.A.B.); vijaytn6@gmail.com (V.M.T.); 2 803, Wing-A, Shrinivas Crossover County, Lagadmala, Sinhgad Road, Near Lokmat Press, Pune 411 041, India; 3 Niels Bohr Institute, University of Copenhagen, Jagtvej 155 A, DK-2200 Copenhagen, Denmark

**Keywords:** chemical kinetics, conservatively perturbed equilibrium, Lyapunov thermodynamic stability, entropy production, Lyapunov direct method

## Abstract

The thermodynamic stability of the evolution of a simple conservatively perturbed chemical equilibrium, that is, a two-step reversible reaction, is investigated using the Lyapunov thermodynamic stability formulation. We show that such systems are *asymptotically thermodynamically stable*.

## 1. Introduction

In our recent publication [1], we described our thermodynamic theory, named Lyapunov thermodynamic stability (LTS), for analyzing the stability of irreversible processes on a generalized footing. This theory uses all the ingredients of Lyapunov’s second method of stability of motion [2,3,4]. The needed Lyapunov function is defined via the rate of entropy production, which is an inseparable property of irreversibility. This theory has been used to establish thermodynamic stability aspects of various irreversible processes, including some of industrial importance [5,6,7,8,9,10,11,12,13].

Recently, a new chemical kinetics tool has been developed, named conservatively perturbed equilibrium (CPE) [14,15,16]. In it, a system is allowed to attain chemical equilibrium. The concentrations of some of the reacting species are then conservatively perturbed while keeping the temperature, pressure, and concentration of at least one chemically reacting species unaltered, and its progress toward the same equilibrium is followed. It is observed that the concentrations of unperturbed species pass through extrema (minima or maxima) before finally attaining their original equilibrium concentrations. It is precisely this extremal out-of-equilibrium feature at some point along the equilibration that makes CPE interesting in practical situations.

A priori, it is possible that the production of such extrema in a CPE evolution makes the corresponding trajectory thermodynamically unstable. To investigate this possibility, we performed an LTS stability analysis of the simplest CPE system. Our results show that such perturbed trajectories are not merely *thermodynamically stable* but are *asymptotically thermodynamically stable*.

## 2. Evolution of Conservatively Perturbed Equilibrium and the Rate of Entropy Production

For simplicity in our presentation, we consider the following two-step reversible consecutive reaction scheme:

(1) $$A\overset{k_{1}}{\underset{k_{-1}}{⇌}}B\overset{k_{2}}{\underset{k_{-2}}{⇌}}C,$$

where A, B, and C are the chemically reactive species and $\left\{k_{i}\right\}$ are the respective rate constants. Thus, the rates of reaction in terms of the respective extent of the advancement of reaction, $\left\{\xi_{i}\right\}$, are

(2) $$\frac{d\xi_{1}}{dt}=k_{1}A-k_{-1}B=-\frac{dA}{dt}$$

(3) $$\frac{d\xi_{2}}{dt}=k_{2}B-k_{-2}C=\frac{dC}{dt},$$

i.e., the number of moles of A converted to B for the first reaction, and the number of moles of B converted to C for the second reaction since the start of the experiment (t=0) (see, for example, [17,18]).

Therefore, we have,

(4) $$\frac{d\xi_{1}}{dt}-\frac{d\xi_{2}}{dt}=k_{1}A-\left(k_{-1}+k_{2}\right)B+k_{-2}C=\frac{dB}{dt}.$$

**Figure 1:**
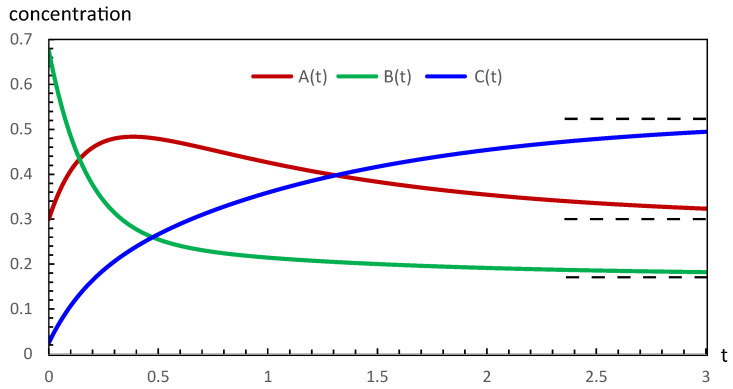
The evolution of the conservatively perturbed reaction in Equation (1) for $k_{1}=1.75\;s^{-1}$, $k_{-1}=3.00\;s^{-1}$, $k_{2}=1.50\;s^{-1}$, $k_{-2}=0.50\;s^{-1}$, $A^{e}=0.30\;M$, $B^{e}=0.175\;M$, and $C^{e}=0.525\;M$. On choosing the concentration of A to be unperturbed initially and the magnitude of the conservative perturbation of B and C to be equal to ±0.50 M, the zero-time ($t_{0}$) concentrations of the three species are $A(t_{0})=0.30\;M$, $B(t_{0})=0.675\;M$, and $C(t_{0})=0.025\;M$. The evolution of the system to the final equilibrium follows the rate expressions in Equations (2)–(4). The observed variations of the concentrations are such that the concentration of B decreases and that of C increases, whereas that of A passes through a maximum value. The equilibrium values $A^{e}$, $B^{e}$, and $C^{e}$ are marked with dashed lines.

**Figure 2:**
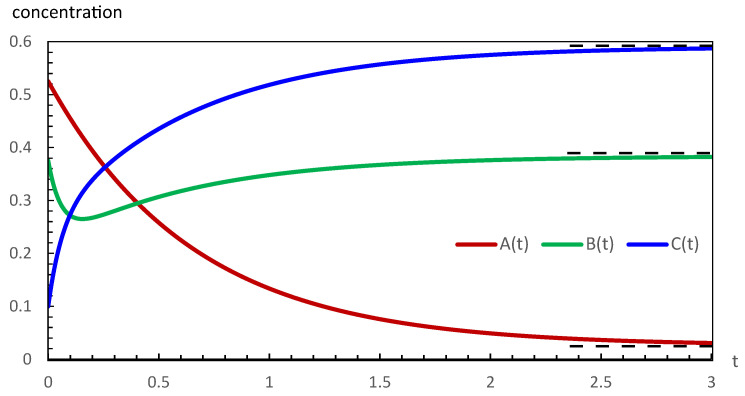
The evolution of the conservatively perturbed reaction in Equation (1) for $k_{1}=1.50\;s^{-1}$, $k_{-1}=0.10\;s^{-1}$, $k_{2}=10.0\;s^{-1}$, $k_{-2}=6.25\;s^{-1}$, $A^{e}=0.025\;M$, $B^{e}=0.375\;M$, and $C^{e}=0.600\;M$. On choosing the concentration of B to be unperturbed initially and the magnitude of the conservative perturbation of A and C equal to ±0.50 M, the initial ($t_{0}$) concentrations of the three species are $A(t_{0})=0.525\;M$, $B(t_{0})=0.375\;M$, and $C(t_{0})=0.100\;M$. The evolution of the system to the final equilibrium follows the rate expressions in Equations (2)–(4). The observed variations of the concentrations are such that the concentration of A decreases and that of C increases, whereas that of B passes through a minimum value. The equilibrium values $A^{e}$, $B^{e}$, and $C^{e}$ are marked with dashed lines.

The respective concentrations are also denoted by A, B, and C in the above rate expressions. In the case of the chemical reaction in Equation (1), the CPE has been studied in two ways [15]. In the first case, A starts at its equilibrium concentration $A^{e}$, and the concentrations of B and C are perturbed under the condition ΔB=−ΔC. In the second case, B starts at its equilibrium concentration $B^{e}$ with perturbations ΔA=−ΔC. The plots of the respective evolutions to the final equilibrium states in the above two cases are shown in Figure 1 and Figure 2.

Throughout this paper, standard SI units are used for the numerical calculations. Thus, time is shown in sec, concentrations in M (molar), affinities in J/mol, and entropy production rates in W/K.

The variation of the concentration A in Figure 1 tells us that initially, the first step proceeds from right to left (implying $d\xi_{1}/dt<0$), and beyond the maximum, the step reverses direction (implying $d\xi_{1}/dt>0$). The second step throughout proceeds from left to right (implying $d\xi_{2}/dt>0$).

In the case of the evolution in Figure 2, at the initial stages, the decrease in the concentration of A is less steep than the increase in the concentration of C. This means that the rate of production of B in the first step of the reaction is exceeded by a much more rapid consumption of B in the second step. Therefore, both steps of the reaction in Equation (1) throughout proceed from left to right, implying $d\xi_{1}/dt>0$ and $d\xi_{2}/dt>0$.

A consecutive two-step reversible reaction has the following rate of entropy production $\Sigma_{S}$ [19,20,21]:

(5) $$\Sigma_{S}=\frac{A_{1}}{T}\frac{d\xi_{1}}{dt}+\frac{A_{2}}{T}\frac{d\xi_{2}}{dt}>0,$$

where *T* is the temperature of the system and $A_{i}$ are the respective chemical affinities, which have the following standard expressions in the present case:

(6) $$A_{1}=\mu_{A}-\mu_{B},\;A_{2}=\mu_{B}-\mu_{C},$$

where $\mu_{i}$ are the respective chemical potentials. The overall positive sign of (5) is guaranteed by the second law of thermodynamics [18]. The thermodynamic expressions of chemical affinities, assuming ideality of the reaction mixture, are

(7) $$\frac{A_{1}}{T}=\frac{A_{1}^{⦵}}{T}-R\ln\left(\frac{B}{A}\right),\;\frac{A_{2}}{T}=\frac{A_{2}^{⦵}}{T}-R\ln\left(\frac{C}{B}\right),$$

where the superscript ⦵ denotes the chosen standard state and *R* is the universal gas constant. Since at equilibrium, chemical affinities are equal to zero, this provides the following expressions for standard-state chemical affinities:

(8) $$\frac{A_{1}^{⦵}}{T}=R\ln\left(\frac{B^{e}}{A^{e}}\right)=R\ln\left(\frac{k_{1}}{k_{-1}}\right),\;\frac{A_{2}^{⦵}}{T}=R\ln\left(\frac{C^{e}}{B^{e}}\right)=R\ln\left(\frac{k_{2}}{k_{-2}}\right).$$

Therefore, Equation (5), on using the expressions of Equations (2), (3), (7), and (8), becomes

(9) $$\Sigma_{S}=R\left(k_{1}A-k_{-1}B\right)\ln\left(\frac{k_{1}A}{k_{-1}B}\right)+R\left(k_{2}B-k_{-2}C\right)\ln\left(\frac{k_{2}B}{k_{-2}C}\right)>0.$$

Notice that each term of Equation (9) is individually positive, since when $\left(k_{1}A-k_{-1}B\right)>0$, we have $\ln\left(\frac{k_{1}A}{k_{-1}B}\right)>0$, and when $\left(k_{1}A-k_{-1}B\right)<0$, we have $\ln\left(\frac{k_{1}A}{k_{-1}B}\right)<0$. The same argument applies to the second term of Equation (9). Therefore, $\Sigma_{S}$ is never negative at any point in the evolution of the CPE.

**Figure 3:**
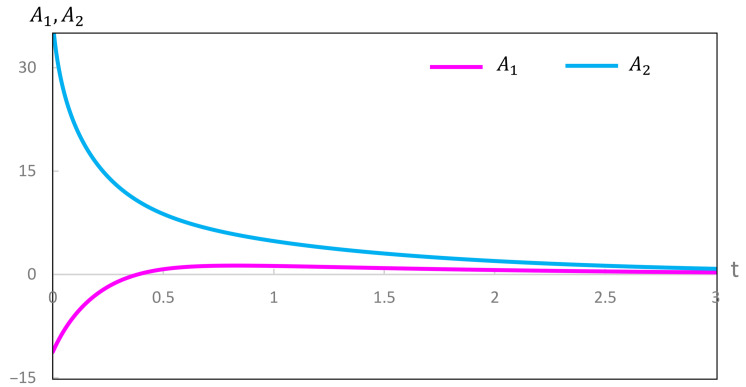
Variation of the chemical affinities $A_{1}$ and $A_{2}$ during the evolution depicted in Figure 1.

**Figure 4:**
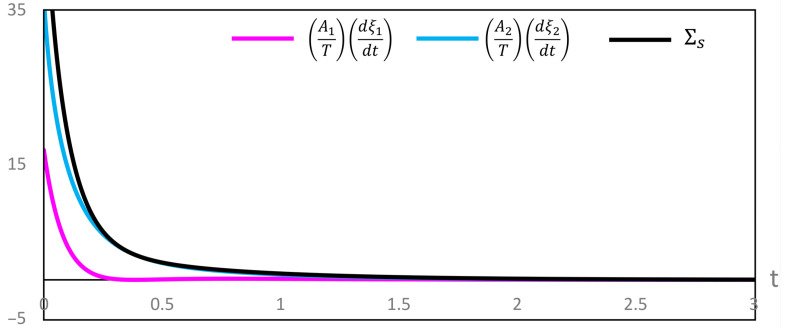
Variation of the contributions to the rate of entropy production and their sum $\Sigma_{S}$ during the evolution depicted in Figure 1.

**Figure 5:**
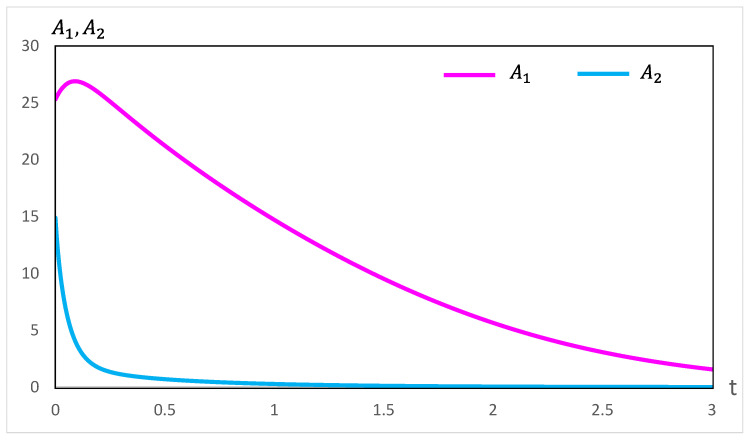
Variation of the chemical affinities $A_{1}$ and $A_{2}$ during the evolution depicted in Figure 2.

**Figure 6:**
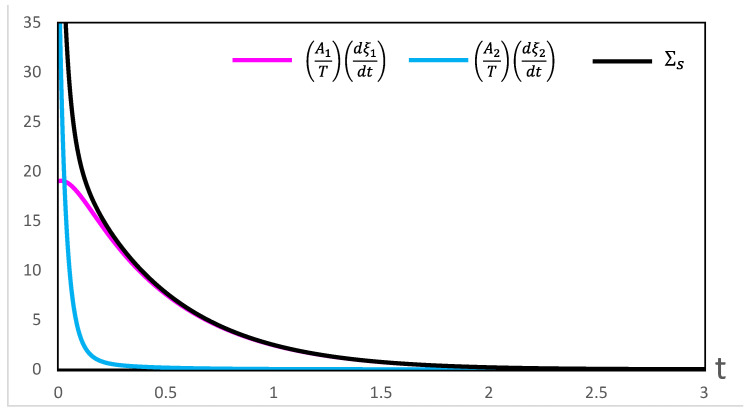
Variation of the contributions to the rate of entropy production and their sum $\Sigma_{S}$ during the evolution depicted in Figure 2.

Our computations of thermodynamic properties corresponding to the evolutions depicted in Figure 1 and Figure 2 are presented in Figure 3, Figure 4, Figure 5 and Figure 6. All calculations are carried out at T=300 K. It can be seen in Figure 3 that the variation of the chemical affinities of the two steps is commensurate with the respective behavior of the two steps depicted in Figure 1. That is, the chemical affinity of the second step, $A_{2}$, remains positive throughout and continuously decreases (this step maintains the direction left to right throughout). In contrast, the chemical affinity of the first step, $A_{1}$, is initially negative (the initial direction of this step is right to left), becomes zero at the maximum of A, and then becomes positive (the direction of the step becomes left to right). Both chemical affinities finally become zero on attaining chemical equilibrium. In spite of this change of direction, Figure 4 illustrates that both rates of entropy production, $\left(A_{1}/T\right)\left(d\xi_{1}/dt\right)$ and $\left(A_{2}/T\right)\left(d\xi_{2}/dt\right)$, remain positive throughout, decreasing eventually to zero. Therefore, the sum of these two terms, that is $\Sigma_{S}$, also remains positive throughout and continuously decreases, as expected. A similar explanation follows for Figure 5 and Figure 6.

Thus, we see that each step of the reaction in Equation (1) during the evolution depicted in Figure 1 and Figure 2 follows the thermodynamically favorable direction with positive entropy production, as set out in Equation (5). This means that the steps remain thermodynamically uncoupled under the conservatively perturbed evolution, i.e., at no point does one reaction drive the other.

## 3. Lyapunov Thermodynamic Stability of the Evolution of Conservatively Perturbed Equilibrium

Lyapunov thermodynamic stability (LTS) theory uses a sign-definite thermodynamic Lyapunov function, $L_{S}$ (refer to, for example, [1]), to analyze the nature of the thermodynamic stability of an irreversible process. The steps of the adopted approach are as follows:

1. Effect a sufficiently small perturbation at a desired time ($t_{0}$) on the trajectory of an evolution whose stability is under investigation.
2. As a result of this perturbation, the system follows a perturbed trajectory.
3. Follow the course of the perturbed trajectory under the same conditions as for the unperturbed one. If within a reasonably short time it reverts to the unperturbed trajectory, the latter is called *asymptotically thermodynamically stable*; if eventually it ends up only in the close vicinity of the unperturbed one, then the latter is termed *thermodynamically stable*; and if the perturbed trajectory diverges away from the unperturbed one, then the latter is established as a *thermodynamically unstable* motion.
4. Along the lines of the Lyapunov second method, these conclusions in LTS are arrived at via the signs of $L_{S}$ and its time derivative, $\dot{L_{S}}$. The basic requirement of $L_{S}$ is that it should depend on the perturbation coordinates and time, as well as vanish *only* on the unperturbed trajectory (the latter is also called the origin). If $\dot{L_{S}}$ is of the opposite sign (i) and vanishes only at the origin, then the latter is obtained as *asymptotically thermodynamically stable*, or else (ii) if it vanishes without reaching the origin, then the latter is established as a *thermodynamically stable motion*. In the case of *thermodynamically unstable motion*, we have the same signs of the Lyapunov function and its time derivative.

### 3.1. Thermodynamic Lyapunov Function of LTS and Stability Criteria

The mathematical expression for $L_{S}$ in LTS is chosen as

(10) $$L_{S}=\Sigma_{S}-\Sigma_{S}^{0}>0,$$

where $\Sigma_{S}>0$ is the rate of entropy production on the perturbed trajectory and $\Sigma_{S}^{0}>0$ is that on the unperturbed trajectory. We denote the quantities pertaining to the unperturbed trajectory by the superscript 0. Recall that the second law of thermodynamics guarantees the positive definite sign of the rate of entropy production. One can refer to $L_{S}>0$ as the *perturbation gain of entropy production* or *excess entropy production* due to perturbation. It is possible that upon perturbation, the rate of entropy production decreases instead of the increase shown in Equation (10). If this occurs, all the arguments about stability here and below, assuming $L_{S}>0$, can be directly converted to $L_{S}<0$, with all subsequent inequalities reversed, as long as its time derivative, $\dot{L_{S}}$, also changes its sign, i.e. becomes positive, since the requirement for Lyapunov stability is that the product $L_{S}\times \dot{L_{S}}$ is negative.

The rate of entropy production depends on the thermodynamic variables appearing in the corresponding Gibbs relation, such as *T*, *p*, and the composition variables. Alternatively, one can use the generalized fluxes as the thermodynamic variables. Let the thermodynamic variables be denoted by $\left\{y_{i}\right\}$ on the perturbed trajectory and by $\left\{y_{i}^{0}\right\}$ on the unperturbed trajectory. The perturbation coordinates $\left\{\alpha_{i}\left(t\right)\right\}$ are defined as

(11) $$\alpha_{i}\left(t\right)=y_{i}\left(t\right)-y_{i}^{0}\left(t\right)\;\left(i=1,2,3,\ldots ,n\right)$$

with the condition $∥\alpha_{i}∥\leq \varepsilon >0$, where ε indicates a sufficiently small neighborhood about the origin. Therefore, the unperturbed trajectory is expressed as

(12) $$\alpha_{i}^{0}\left(t\right)=y_{i}^{0}\left(t\right)-y_{i}^{0}\left(t\right)=0\;\left(i=1,2,3,\ldots ,n\right)$$

with the condition $∥\alpha_{i}^{0}∥\leq \kappa >0$, where κ<ε indicates another sufficiently small neighborhood about the origin for the initial perturbation.

Let the differential equations on the unperturbed trajectory be

(13) $$\frac{dy_{i}^{0}\left(t\right)}{dt}=f_{i}^{0}\left(\left\{y_{j}^{0}\left(t\right)\right\}\right)\;\left(i,j=1,2,3,\ldots ,n\right)$$

and those on the perturbed trajectory be

(14) $$\frac{dy_{i}\left(t\right)}{dt}=f_{i}\left(\left\{y_{j}\left(t\right)\right\}\right),\;\left(i,j=1,2,3,\ldots ,n\right).$$

The differential equations governing the perturbation coordinates, from Equations (11)–(14), are

(15) $$\frac{d\alpha_{i}\left(t\right)}{dt}=F_{i}\left(\{\alpha_{i}\left(t\right)\}\right)\;\left(i=1,2,3,\ldots ,n\right).$$

Thus, for a non-autonomous system, we have the following functional dependence:

(16) $$L_{S}\left(t\right)=L_{S}\left(\{\alpha_{i}\left(t\right)\},t\right)>0.$$

Notice that $L_{S}$ vanishes on the unperturbed trajectory, which, from Equations (10) and (12), gives

(17) $$L_{S}\left(t\right)=L_{S}\left(\{0\},t\right)=0.$$

Next, the time derivative of $L_{S}$ is

(18) $$\dot{L_{S}}=\frac{dL_{S}}{dt}=\frac{\partial L_{S}}{\partial t}+\sum_{i}\frac{\partial L_{S}}{\partial \alpha_{i}}\frac{d\alpha_{i}}{dt}.$$

Therefore, along the lines of the Lyapunov second method for the stability of motion [2,3,4], we have the following thermodynamic stability criteria:

1. *Thermodynamic stability* is expressed as
  (19) $$\begin{array}{c} L_{S}\left(\{\alpha_{i}\left(t\right)\},t\right)>0\;\;\forall \left\{\alpha_{i}\left(t\right)\right\}\in D\setminus \left\{0\right\}\\ \dot{L_{S}}=\frac{dL_{S}}{dt}=\frac{\partial L_{S}}{\partial t}+\sum_{i}\frac{\partial L_{S}}{\partial \alpha_{i}}\frac{d\alpha_{i}}{dt}\leq 0\;\;\forall \left\{\alpha_{i}\left(t\right)\right\}\in D\setminus \left\{0\right\} \end{array}$$
  That is, $\dot{L_{S}}$ may vanish before the perturbed trajectory reaches the unperturbed one.
2. *Asymptotic thermodynamic stability* is described as
  (20) $$\begin{array}{c} L_{S}\left(\{\alpha_{i}\left(t\right)\},t\right)>0\;\;\forall \left\{\alpha_{i}\left(t\right)\right\}\in D\setminus \left\{0\right\}\\ \dot{L_{S}}=\frac{dL_{S}}{dt}=\frac{\partial L_{S}}{\partial t}+\sum_{i}\frac{\partial L_{S}}{\partial \alpha_{i}}\frac{d\alpha_{i}}{dt}<0\;\;\forall \left\{\alpha_{i}\left(t\right)\right\}\in D\setminus \left\{0\right\} \end{array}$$
  That is, $\dot{L_{S}}$ vanishes *only* on the unperturbed trajectory.

### 3.2. The Basic Expressions for Analyzing the Thermodynamic Stability of the Evolution of Conservatively Perturbed Equilibrium

In the stability analysis, the perturbations have to be sufficiently small; hence, we represent the perturbations in the concentrations as δA, δB, and δC under the condition of conservation of mass given by

(21) δA+δB+δC=0.

The concentrations on the perturbed trajectory are expressed as

(22) $$A=A^{0}+\delta A,\;B=B^{0}+\delta B,\;C=C^{0}+\delta C.$$

Next, using the expressions in Equations (21) and (22) in the rate expressions of Equations (2)–(4), the differential equations for the perturbation coordinates become

(23) $$-\frac{d\delta A}{dt}=k_{1}\delta A-k_{-1}\delta B$$

(24) $$-\frac{d\delta B}{dt}=-\left(k_{1}-k_{-2}\right)\delta A+\left(k_{-1}+k_{2}+k_{-2}\right)\delta B.$$

Notice that in light of the mass balance condition in Equation (21), we have only two independent perturbation coordinates; hence, only two corresponding differential equations are needed.

Now we introduce the perturbations in Equation (22), the mass balance in Equation (21), and the assumption that δA≪A, δB≪B, and δC≪C into the rate of entropy production on the perturbed trajectory, Equation (9), to obtain

(25) $$\begin{array}{cc} \Sigma_{S}= & R\left(k_{1}A^{0}-k_{-1}B^{0}+k_{1}\delta A-k_{-1}\delta B\right)\times \left[\ln\left(\frac{k_{1}A^{0}}{k_{-1}B^{0}}\right)+\left(\frac{\delta A}{A^{0}}-\frac{\delta B}{B^{0}}\right)\right]\\ \; & +R\left(k_{2}B^{0}-k_{-2}C^{0}+k_{-2}\delta A+\left(k_{2}+k_{-2}\right)\delta B\right)\\ & \;\times \left[\ln\left(\frac{k_{2}B^{0}}{k_{-2}C^{0}}\right)+\frac{\delta A}{C^{0}}+\left(\frac{1}{B^{0}}+\frac{1}{C^{0}}\right)\delta B\right]>0. \end{array}$$

Similarly, Equation (9) for the unperturbed trajectory becomes

(26) $$\Sigma_{S}^{0}=R\left(k_{1}A^{0}-k_{-1}B^{0}\right)\ln\left(\frac{k_{1}A^{0}}{k_{-1}B^{0}}\right)+R\left(k_{2}B^{0}-k_{-2}C^{0}\right)\ln\left(\frac{k_{2}B^{0}}{k_{-2}C^{0}}\right)>0.$$

Therefore, the expression for $L_{S}$, as defined in Equation (10), is obtained from Equations (25) and (26), after a little algebra, as

(27) $$L_{S}=X_{\delta A}^{0}\delta A+X_{\delta B}^{0}\delta B$$

with

(28) $$\begin{array}{cc} X_{\delta A}^{0}= & R\left[k_{1}\ln\left(\frac{k_{1}A^{0}}{k_{-1}B^{0}}\right)+k_{-2}\ln\left(\frac{k_{2}B^{0}}{k_{-2}C^{0}}\right)\right.\\ & \left.+\left(\frac{k_{1}A^{0}-k_{-1}B^{0}}{A^{0}}\right)+\left(\frac{k_{2}B^{0}-k_{-2}C^{0}}{C^{0}}\right)\right] \end{array}$$

and

(29) $$\begin{array}{cc} X_{\delta B}^{0}= & R\left[\left(k_{2}+k_{-2}\right)\ln\left(\frac{k_{2}B^{0}}{k_{-2}C^{0}}\right)-\left(\frac{k_{1}A^{0}-k_{-1}B^{0}}{B^{0}}\right)\right.\\ & \left.\;-k_{-1}\ln\left(\frac{k_{1}A^{0}}{k_{-1}B^{0}}\right)+\left(k_{2}B^{0}-k_{-2}C^{0}\right)\left(\frac{1}{B^{0}}+\frac{1}{C^{0}}\right)\right]. \end{array}$$

Notice that the coefficients $X_{\delta A}^{0}$ and $X_{\delta B}^{0}$ in Equation (27) have their respective expressions in terms of time-dependent quantities belonging to the unperturbed trajectory. Hence, at constant δA and δB, $L_{S}$ still varies with time. This constitutes a *non-autonomous system* because we have $\partial L_{S}/\partial t\neq 0$.

The time rate of $L_{S}$ from Equation (27), and on using Equations (23) and (24), becomes

(30) $$\begin{array}{cc} \dot{L_{S}}=\frac{dL_{S}}{dt}= & \left[k_{1}\left(X_{\delta B}^{0}-X_{\delta A}^{0}\right)-k_{-2}X_{\delta B}^{0}+\frac{dX_{\delta A}^{0}}{dt}\right]\delta A\\ \; & +\left[\left(X_{\delta A}^{0}-X_{\delta B}^{0}\right)k_{-1}-\left(k_{2}+k_{-2}\right)X_{\delta B}^{0}+\frac{dX_{\delta B}^{0}}{dt}\right]\delta B. \end{array}$$

## 4. Results and Discussion

### 4.1. Plots of $L_{S}$ and $\dot{L_{S}}$ Under Various Choices of Perturbation

We studied the thermodynamic stability aspects of the trajectories depicted in Figure 1 and Figure 2 against sufficiently small perturbations under the mass conservation condition in Equation (21). These small perturbations, applied for the purpose of stability analysis, should not be confused with the larger perturbations of ±0.50 M applied at time t=0 as part of the conservatively perturbed equilibrium (CPE) schedule. The sufficiently small perturbations are superimposed on the CPE. The small perturbations were effected at five different stages (times) of each evolution in Figure 1 and Figure 2. The stages chosen are (a) beginning, (b) left side of the extremum, (c) at the extremum, and (d) and (e) on the right side of the extremum, where the CPE trajectories achieved the concentrations listed as $A^{0},B^{0}$, and $C^{0}$. Due to mass conservation, these small perturbations always add up to zero, δA+δB+δC=0.

**Figure 7:**
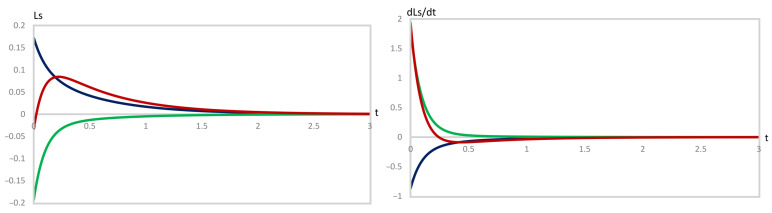
Variation of $L_{S}$ and ${\dot{L}}_{S}$ versus time, *t*, using Equations (27) and (30), respectively, for the perturbation of the evolution in Figure 1, corresponding to the data of the rows named *c* in Table 1, that is, at the extremum of the concentration of A. The signs of the perturbations are δA>0, δB>0, δC<0 (black), δA>0, δB<0, δC>0 (green), and δA>0, δB<0, δC<0 (red). The data represented by the red line has been scaled by a factor of 5 in order to fit in the same plot.

**Figure 8:**
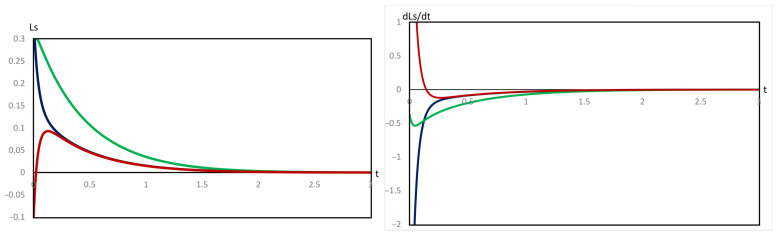
Variation of $L_{S}$ and ${\dot{L}}_{S}$ versus time, *t*, using Equations (27) and (30), respectively, for the perturbation of the evolution in Figure 2, corresponding to the data of the rows named *c* in Table 2, that is, at the minimum of B. The signs of the perturbations are δA>0, δB>0, δC<0 (black), δA>0, δB<0, δC<0 (green), δA>0, δB<0, and δC>0 (red).

The full set of the 30 systems investigated is summarized in Table 1 and Table 2. Out of these, three representative situations, each at the A maximum (Figure 1) and at the B minimum (Figure 2), are presented in Figure 7 and Figure 8. At these points, we focus on cases of perturbation in a Lyapunov sense at times when an extremum is observed in the CPE evolution, i.e., at stages named *c* in the tables. The full set of plots is available on request from Dr. Vijay Tangde <vijaytn6@gmail.com>.

### 4.2. Discussion

The sets of perturbations effected using the mass conservation condition in Equation (21) and studied for the thermodynamic stability of the evolutions presented in Figure 1 and Figure 2 are summarized in Table 1 and Table 2, respectively.

In the case of the evolution in Figure 1, we have the effected perturbations (i) δA(t)>0, δB(t)>0, δC(t)<0, (ii) δA(t)>0, δB(t)<0, δC(t)>0, and (iii) δA(t)>0, δB(t)<0, δC(t)<0. These perturbations were effected before the maximum of A, at the maximum of A, and beyond the maximum of A. Thus, a total of 15 pairs of plots of $L_{S}$ and ${\dot{L}}_{S}$ were generated.

In case (i), for all instances studied, the values of these two functions satisfy $L_{S}>0$ and ${\dot{L}}_{S}<0$, and both vary continuously and finally vanish at the origin. In case (ii), the signs are reversed, $L_{S}<0$ and ${\dot{L}}_{S}>0$, while both still vary continuously and finally vanish at the origin. Thus, in these cases, asymptotic thermodynamic stability is established. Case (iii) is different. It does eventually display asymptotic thermodynamic stability, like cases (i) and (ii), because $L_{S}$ and ${\dot{L}}_{S}$ have opposite signs and finally both vanish at the origin. But, during short initial time periods, $L_{S}$ and ${\dot{L}}_{S}$ have the same sign, creating a temporary state of instability (representative plots of all three cases are depicted in Figure 7).

Notice that along the black and green curves in Figure 7, $L_{S}$ and ${\dot{L}}_{S}$ are of opposite signs, and thus $L_{S}\times {\dot{L}}_{S}<0$ consistently, as required for stability, and they smoothly vanish at large *t* on the unperturbed trajectory. This establishes the asymptotic thermodynamic stability of the unperturbed trajectory against these perturbations. Applying perturbations δA(t)=0.00625, δB(t)=−0.0030, and δC(t)=−0.00325 yields a different behavior. The resulting variations of $L_{S}$ and ${\dot{L}}_{S}$ are depicted as the red curves in Figure 7. During a short initial time period, $L_{S}$ and ${\dot{L}}_{S}$ both change signs, but at slightly different times, and consequently exhibit a brief period of instability.

**Figure 9:**
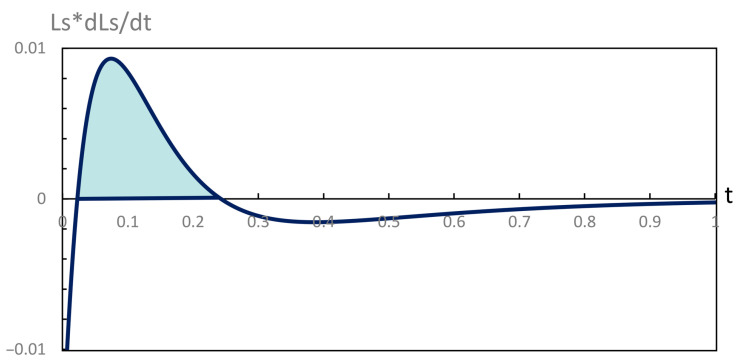
Variation of $L_{S}\times {\dot{L}}_{S}$ versus time, *t*, corresponding to the red curve in Figure 7. The transient instability lies between t=0.01and0.22. On both sides of this time period, this product is negative and eventually vanishes at large *t*.

Subsequently, opposite signs are restored, and the Lyapunov curve continues toward a zero value on the unperturbed trajectory. Thus, during this short initial interval, $L_{S}\times {\dot{L}}_{S}>0$, implying instability, as depicted in light blue in Figure 9. This means that the perturbed trajectory passes through a transient instability during the short initial time period but finally approaches the unperturbed trajectory asymptotically, thereby demonstrating asymptotic thermodynamic stability.

In the case of the evolution in Figure 2, the effected perturbations are (i) δA(t)>0, δB(t)>0, δC(t)<0, (ii) δA(t)>0, δB(t)<0, δC(t)<0, and (iii) δA(t)>0, δB(t)<0, δC(t)>0. These perturbations were effected before the minimum of B, at the minimum of B, and beyond the minimum of B.

In this case, we also generated 15 pairs of plots. For all cases of (i) and (ii), we have $L_{S}>0$ and ${\dot{L}}_{S}<0$ throughout, and both finally vanish at the origin. Hence, against these perturbations, the evolution in Figure 2 is established as asymptotically thermodynamically stable. The black and green curves in Figure 8 are representative plots of cases (i) and (ii) when the perturbation is effected at the minimum of B.

**Figure 10:**
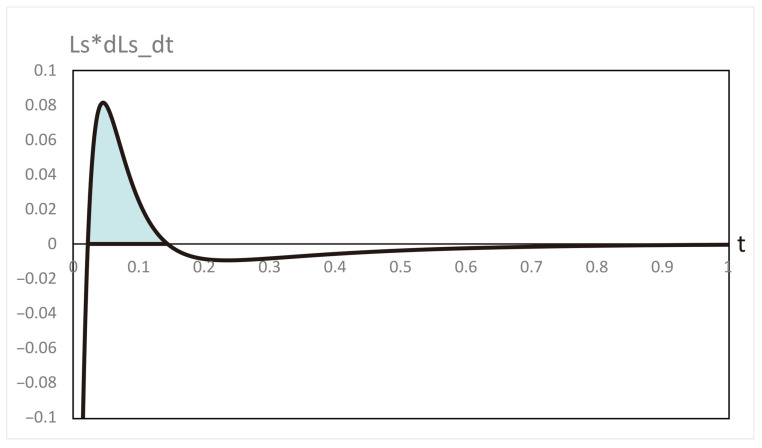
Variation of $L_{S}\times {\dot{L}}_{S}$ versus time, *t*, corresponding to Figure 9. The transient instability lies between t=0.02and0.18. On both sides of this time period, this product is negative and eventually vanishes at large *t*.

Likewise, in case (iii), barring a very short initial time period, we observe that $L_{S}>0$ and ${\dot{L}}_{S}<0$ throughout, and they vanish only at the origin. Hence, the evolution in Figure 2 against the perturbation of type (iii) is established as asymptotically thermodynamically stable. However, in this case, at the initial stages, for a short time period where $L_{S}\times {\dot{L}}_{S}>0$, the system passes through a transient instability. The red curve in Figure 8 shows such a perturbation effected at the minimum concentration of B. The brief period of instability is depicted in Figure 10, analogous to Figure 9.

It is worth emphasizing that the two systems producing transient instability have a common feature. Recall that the CPE evolution in Figure 1 is obtained when the concentration of A is unperturbed, whereas the evolution in Figure 2 is generated when the concentration of B is unperturbed. In both cases, the signs of the perturbations in the concentrations of the respective other two species (δB and δC in the former case, and δA and δC in the latter case), in the Lyapunov sense, are opposite to the signs of the perturbations effected on A (in the former case) and B (in the latter case).

## 5. Conclusions

The overall conclusion is that the evolutions of conservatively perturbed chemical equilibrium in a two-step consecutive chemical reaction are *asymptotically thermodynamically stable* despite producing extrema in the concentration of the unperturbed reactive species.

In general, from a chemical kinetics point of view, a conservatively perturbed setup is equivalent to studying kinetics with, for example, initial concentrations $A^{e},B^{0}$, and $C^{0}$ or $A^{0},B^{e}$, and $C^{0}$, with the respective conditions $B^{0}+C^{0}=B^{e}+C^{e}$ and $A^{0}+C^{0}=A^{e}+C^{e}$. The two cases of CPE investigated in the present work are depicted in Figure 1 and Figure 2. Of course, we can see that in the former case, the first step initially proceeds from right to left, and after the attainment of the maximum concentration of A, it changes its direction, while the second step maintains its direction from left to right throughout. In contrast, in the latter case, despite the observation of a minimum in the concentration of B, both steps maintain their direction from left to right throughout. Despite the observation of extrema in the concentration of unperturbed species and the change of direction of the first step in Figure 1, these CPE evolutions are proven to be asymptotically thermodynamically stable. This conclusion strengthens the thermodynamic notion that such unusual-looking behavior does not influence stability, provided the second law of thermodynamics is not violated.

In future studies, using the same line of reasoning, we will investigate the thermodynamic stability of more complex CPE systems whose chemical kinetics have already been carried out [14,15].

## Figures and Tables

**Table 1:** The set of values of the concentrations on unperturbed trajectories and those of the perturbation coordinates used w.r.t. the evolution in Figure 1. Thus, the CPE perturbation affects B(0) and C(0) by ±0.50 M, while A starts out at its equilibrium concentration.

S. No.	Time, *t*	$A^{0}\left(t\right)$	$B^{0}\left(t\right)$	$C^{0}\left(t\right)$	δA	δB	δC
i	δA>0, δB>0, δC<0						
a	0	0.300	0.675	0.025	0.0030	0.00325	−0.00625
b	0.2	0.458	0.375	0.167			
c	0.4 (At Maximum A)	0.482	0.276	0.242			
d	1.0	0.427	0.214	0.359			
e	2.0	0.350	0.188	0.462			
ii	δA>0, δB<0, δC>0						
a	0	0.300	0.675	0.025	0.0030	−0.00625	0.00325
b	0.2	0.458	0.375	0.167			
c	0.4 (At Maximum A)	0.482	0.276	0.242			
d	1.0	0.427	0.214	0.359			
e	2.0	0.350	0.188	0.462			
iii	δA>0, δB<0, δC<0						
a	0	0.300	0.675	0.025	0.00625	−0.0030	−0.00325
b	0.2	0.458	0.375	0.167			
c	0.4 (At Maximum A)	0.482	0.276	0.242			
d	1.0	0.427	0.214	0.359			
e	2.0	0.350	0.188	0.462			

**Table 2:** The set of values of the concentrations on unperturbed trajectories and those of the perturbation coordinates used w.r.t. the evolution in Figure 2. Thus, the CPE perturbation affects A(0) and C(0) by ±0.50 M, while B starts out at its equilibrium concentration.

S. No.	Time, *t*	$A^{0}\left(t\right)$	$B^{0}\left(t\right)$	$C^{0}\left(t\right)$	δA	δB	δC
i	δA>0, δB>0, δC<0						
a	0	0.525	0.375	0.100	0.00225	0.003	−0.00525
b	0.05	0.490	0.298	0.212			
c	0.15 (At Minimum B)	0.416	0.265	0.319			
d	1.0	0.135	0.338	0.527			
e	2.0	0.062	0.375	0.563			
ii	δA>0, δB<0, δC<0						
a	0	0.525	0.375	0.100	0.00525	−0.00225	−0.003
b	0.05	0.490	0.298	0.212			
c	0.15 (At Minimum B)	0.416	0.265	0.319			
d	1.0	0.135	0.338	0.527			
e	2.0	0.062	0.375	0.563			
iii	δA>0, δB<0, δC>0						
a	0	0.525	0.375	0.100	0.00225	−0.00525	0.003
b	0.05	0.490	0.298	0.212			
c	0.15 (At Minimum B)	0.416	0.265	0.319			
d	1.0	0.135	0.338	0.527			
e	2.0	0.062	0.375	0.563			

## Data Availability

All 60 detailed time evolution graphs are available on request from Vijay Tangde <vijaytn6@gmail.com>.

## Abbreviations

The following abbreviations are used in this manuscript:

LTS	Lyapunov Thermodynamic Stability
CPE	Conservatively Perturbed Equilibrium
